# Lung Ultrasound Findings in COVID-19: A Descriptive Retrospective Study

**DOI:** 10.7759/cureus.23375

**Published:** 2022-03-21

**Authors:** Talib Omer, Collin Cousins, Taylor Lynch, Nhu-Nguyen Le, Dana Sajed, Thomas Mailhot

**Affiliations:** 1 Emergency Department, Los Angeles County+University of Southern California Medical Center, Los Angeles, USA

**Keywords:** prognostic markers, covid-19, lung ultrasound (lus), shred sign, b-lines, bedside ultrasound, pocus, point-of-care-ultrasound, ultrasonography, ultrasound

## Abstract

Background

Point-of-care ultrasound (POCUS) is an indispensable tool in emergency medicine. With the emergence of the coronavirus disease 2019 (COVID-19) pandemic caused by severe acute respiratory syndrome coronavirus 2 (SARS-CoV-2), a need for improved diagnostic capabilities and prognostic indicators for patients who are symptomatic for COVID-19 has become apparent. POCUS has been demonstrated to be a useful diagnostic and prognostic tool in the emergency department (ED) in assessing other lung complications. Still, limited data regarding its utility in assessing COVID-19 are available. This study sought to evaluate whether POCUS findings in the ED were correlated with vital signs or laboratory abnormalities typically seen among patients with COVID-19.

Methods

A retrospective study was conducted that included 39 patients who presented with COVID-19 and systemic inflammatory response syndrome (SIRS) to a large, urban tertiary care ED. The study population was limited to adults aged 18 and above who came to the ED with the primary complaint of respiratory symptoms, met SIRS criteria on admission, and had images of at least one anterior and one posterior intercostal space per lung and a minimum of four intercostal spaces. POCUS images were obtained by trained operators in the ED using portable ultrasound machines, recorded in an image database, and reviewed by ultrasound fellowship-trained emergency physicians. Clinical data (e.g., acute phase reactants and vital signs) were obtained through a chart review of patients’ electronic medical records.

Results

Both the percentage of intercostal spaces with B-lines and the percentage of merging B-lines were correlated with decreased oxygen saturation on presentation. No other statistically significant correlations were observed between these sonographic findings and other vital signs or acute phase reactants, nor between these clinical data and the percentage of intercostal spaces that were positive for the shred sign.

Conclusions

With the emergence of the COVID-19 pandemic, emergency medicine physicians are on the frontline of identifying and caring for patients affected by the virus. This study found that sonographic findings associated with interstitial pneumonitis, notably merging B-lines, and the overall percentage of intercostal spaces with B-lines, were clearly associated with worsening oxygen saturation, now thought to be one of the driving causes of morbidity and mortality in COVID-19. As ultrasound has become a ubiquitous and indispensable tool in the ED, this study demonstrated its utility in assessing and managing patients with COVID-19. Bedside ultrasound is a cheap, fast, and non-invasive tool that healthcare providers can use as an essential adjunct in addition to laboratory markers and other imaging modalities for the diagnosis and prognosis of COVID-19.

## Introduction

Point-of-care ultrasound (POCUS) has become an indispensable tool of the emergency medicine physician over the last several decades. With the emergence of the coronavirus disease 2019 (COVID-19) pandemic caused by severe acute respiratory syndrome coronavirus 2 (SARS-CoV-2) has come a push for improved diagnostic capabilities and prognostic indicators for patients with symptomatic COVID-19. POCUS has been demonstrated to be a useful diagnostic and prognostic tool in the emergency department (ED) in assessing lung consolidation, pneumothorax, and pulmonary edema [1-4]. Of the many deleterious effects that COVID-19 has on the body, one is acute interstitial pneumonitis, which can lead to hypoxia, respiratory failure, and ultimately death in an unfortunately high number of patients [5]. POCUS is a fast and effective bedside tool that is already used effectively to evaluate patients with interstitial pneumonia [1]. Therefore, it would seem as though POCUS could be an important modality in the evaluation of patients with known or suspected COVID-19. However, there are limited data so far regarding the utility of POCUS in assessing COVID-19.

Current research and background

The research regarding modalities used to diagnose COVID-19 shows that reverse transcription-polymerase chain reaction (RT-PCR) and chest x-ray (CXR) both have sensitivities of 59% versus computed tomography (CT) of the thorax that has been found to have a sensitivity of 88% [6,7]. The *New England Journal of Medicine *however estimates the sensitivity of the RT-PCR to be around 70%, still below the sensitivity of the CT scan [8]. There is no current gold standard for diagnosing COVID-19 as the scientific community is just beginning to produce a body of literature on the subject. Although useful in determining the severity of illness, CT is not always feasible from an economic or practical point of view in the ED due to costs and delays associated with decontamination. However, there is strong evidence in the literature demonstrating a high level of accuracy of lung ultrasound in detecting many of the findings in patients with COVID-19. Ground glass opacities and interstitial lung disease, seen with CT, are manifest by the number and degree of B-lines [4], a vertical linear artifact seen on lung ultrasound that extends from the pleura through the far field of the image [9]. These artifacts are associated with a number of pathological findings, including pneumonia, cardiogenic pulmonary edema, interstitial lung disease, acute respiratory distress syndrome and pulmonary fibrosis [10]. A small retrospective study out of Xi'an Chest Hospital, China, during January and February 2020 analysed the lesion type found on ultrasound in 20 patients found to be COVID-19 positive [11]. This paper was the first published literature on the use of ultrasound in COVID-19 patients and found that a large number of B-lines, subpleural consolidations and subpleural pulmonary consolidation were the most common findings of POCUS. Additionally, Peng et al. also analysed the utility of POCUS in 20 patients afflicted with COVID-19 out of China and found similar results regarding the thickening of the pleural line with irregularities along the border, B lines (in a focal, multifocal, and confluent pattern), as well as various consolidation patterns with occasional mobile air bronchograms. Both of these studies, although helpful, were limited in correlating ultrasound findings with any vital sign or laboratory abnormalities [12,13]. Therefore, our study set out to evaluate whether POCUS findings in the ED were correlated with vital signs or laboratory abnormalities or additionally could provide prognostic information regarding patients with COVID-19.

## Materials and methods

Population

This study evaluated 39 patients with the primary complaint of respiratory symptoms who presented to a large, urban, tertiary care ED in Los Angeles from May 1 to June 30, 2020, and who subsequently tested positive for COVID-19. The 39 patients met systemic inflammatory response syndrome (SIRS) criteria on admission and included 33 men and 6 women ranging in age between 23 and 77 years, with a mean age of 49.2 years. Due to the fact that this was a small study population, the inclusion criteria used to obtain the sample of 39 patients were narrowed to limit confounding variables.

SIRS criteria was defined as having two or more of the following: temperature >38 or <35°C, heart rate (HR) >90 bpm, respiratory rate >20, white blood cell count (WBC) >12,000 or <4,000 or having >10% bands. Vital signs were assessed in triage as well as when patients were roomed in the emergency room. With the exception of WBC as a possible inclusion criterion, patients were not included or excluded based on any other laboratory parameters.

POCUS images were obtained by the treating physicians who were either board-certified emergency medicine physicians or PGY-2/PGY-3 residents who had completed at least a two-week ultrasound rotation. All images were obtained using the same portable SonoSite Edge II ultrasound machines (Fujifilm SonoSite, Bothell, WA) in either ‘Cardiac’ or ‘Abdominal’ setting, using the curvilinear (c60) or phased array transducer (p19). Images were obtained shortly after the patient arriving to the emergency room. Patients were only included in the study if they were determined to have adequate POCUS examinations per our departmental COVID lung protocol, defined as containing images of at least one anterior and one posterior intercostal space per lung.

Study characteristics

The study was submitted for review by the institutional review board of the University of Southern California who determined this study to be exempt from the requirements for continuing review (HS-20-00551). All eligible study subjects with a positive SARS-CoV-2 RT-PCR result were identified and then further narrowed down to the group of subjects for whom adequate POCUS images were recorded and available for review in the emergency department’s POCUS image database (QPath; Telexy, Seattle, WA). An analysis of the clinical data was then conducted through chart review of the hospital’s electronic medical record (EMR). The acquired ultrasound images were reviewed by ultrasound fellowship-trained emergency physicians, who were blinded to the patients’ clinical data and outcome.

**Figure 1:**
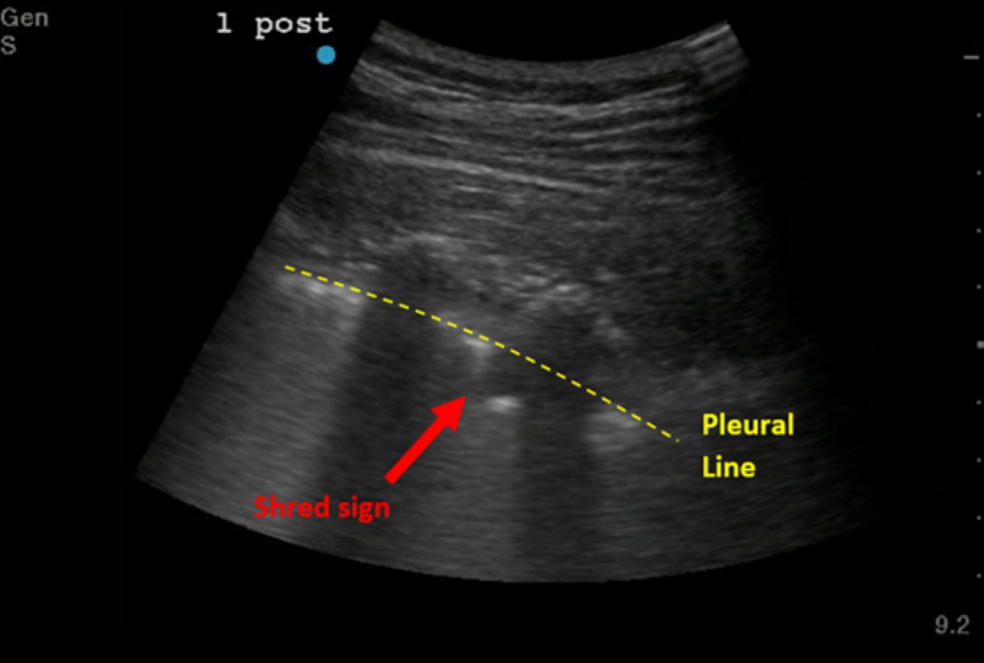
‘Shred sign’, also known as fractal sign (labeled with an arrow) and pleural line (labeled with a dotted line) Scale on the right: each dot equals 1 cm of tissue depth.

**Figure 2:**
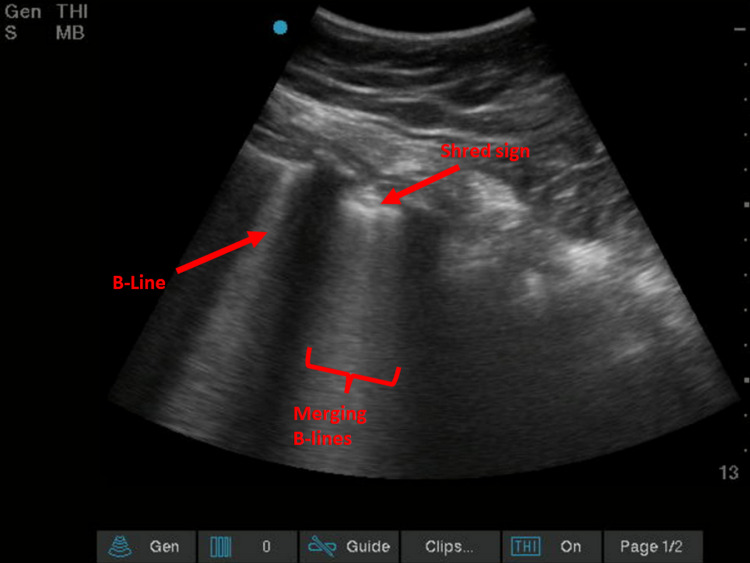
Singular B-line (labeled), merging B-lines (labeled) and subtle ‘shred sign’ (labeled) Scale on the right: each dot equals 1 cm of tissue depth.

POCUS images were assessed for the percentage of intercostal spaces that had B-lines out of the total amount of intercostal spaces visualized and the percentage of merging B-lines within those intercostal spaces. Merging was defined as confluent B-lines (Figure 1). Images were also assessed for the presence of a ‘shred sign,’ a sonographic sign of small subpleural lung consolidation characterized by an irregular pleural margin (Figure 2). This was then further broken down into the percentage of lung spaces that demonstrated this finding, again only assessed in patients in which a minimum of four intercostal spaces were assessed. The number of intercostal spaces assessed varied among patients and was dependent on the number of adequate views the operator was able to obtain. Among the 39 patients in the study, the number of adequate views of intercostal spaces obtained by the operator ranged between 6 and 27.

Outcomes

As COVID-19 has a relatively high mortality and morbidity leading to vital sign and laboratory abnormalities compared to other seasonal viruses, the data were analysed to evaluate how POCUS findings correlated with vital signs and lab values. In the limited data that have already emerged regarding COVID-19, there appear to be a number of laboratory markers, specifically acute phase reactants C-reactive protein (CRP), D-dimer and ferritin, which are correlated with patient outcomes [11]. This study investigated how POCUS findings correlated with these same acute phase reactants, CRP, D-dimer and ferritin, as well as procalcitonin and lactate dehydrogenase (LDH). Additionally, the correlation between POCUS findings and vital signs of respiratory rate, HR, and oxygen saturation on presentation was examined.

## Results

This study enrolled 39 patients 23-77 years of age with a mean age of 49.2 years (Table 1). The majority of patients were men (n = 33, 84.6%), had one or more comorbidity (n = 28, 71.8%), and had fever (n = 23, 59.0%). The most common comorbidities were hypertension (n = 14, 35.9%), diabetes mellitus (n = 10, 25.6%), and obesity (n = 6, 15.4%). Three patients (7.6%) had a history of congestive heart failure but none with an acute exacerbation. Only four of seven patients (17.9%) were intubated and five (12.8%) died as a result of their infection.

**Table 1:** Demographics of study participants

	n		%
Female	6		15.4
Comorbidity			
Yes	28		71.8
No	11		28.2
Intubated			
Yes	7		17.9
No	32		82.1
Deceased			
Yes	5		12.8
No	34		87.2
	Mean	SD	Range
Age (years)	49.2	13.3	23–77
No. of comorbidities	1.9	1.9	0–7
Temperature (°C)	38.1	1.2	34.7–40.0
Weight (kg)	85.6	20.7	62–135

The first characteristic assessed was how the presence of the shred sign, a sonographic sign of lung consolidation, related to levels of acute phase reactants and vital signs as measured on presentation to the ED (Table 2). No statistically significant correlations were found between the percentage of intercostal spaces that showed a shred sign and any acute phase reactant or vital sign (Table 3).

**Table 2:** Data for all patients RR; respiratory rate; HR, heart rate; CRP, C-reactive protein; LDH, lactate dehydrogenase

Patient no.	Vital signs			Inflammatory laboratory markers					Ultrasound outcomes		
	RR	HR	SpO_2_ (on presentation)	Procalcitonin	LDH	D-dimer	CRP	Ferritin	% B-lines	% Shred sign	% Merging B-lines
1	21	88	96	0.23	551	0.78	292	3992	66.67	25.00	25.00
2	22	88	40	0.37	279	2.27	63	712	88.89	22.22	22.22
3	37	122	69	0.02	362	2.83	62.4	154	92.86	42.86	57.14
4	24	97	92	0.37	452		191	692	66.67	27.78	27.78
5	22	108	91	0.65	399	0.64	195	790	55.56	50.00	22.22
6	37	106	89	2.18	400	4.99	115	2310	94.12	35.29	64.71
7	37	92	94	0.11	292	0.66	188	715	76.47	47.06	58.82
8	24	139	91	0.19	489	0.64	210	1115	40.00	0.00	10.00
9	16	119	90	0.3	488	1.15	278	684	92.31	30.77	7.69
10	40	100	85	0.22	376	1.46	101.5	701	92.86	14.29	21.43
11	28	140	81	1.61	784	0.84	168	2377	84.62	15.38	23.08
12	30	94	87	0.53	432	0.7	92.4	3269	76.19	23.81	14.29
13	24	159	83	0.1	540	2.44	81	586	54.55	18.18	0.00
14	39	114	85	100	818	12.25	241	5879	90.91	27.27	36.36
15	34	125	95	0.15	182	0.3	199	213	100.00	0.00	33.33
16	52	110	91	0.47	401	0.57	194	921	60.00	20.00	10.00
17	28	111	91	0.08	281		34		88.89	27.78	11.11
18	31	78	95	0.08	419	0.49	74.5	536	60.00	5.00	35.00
19	18	118	96	0.67	339	3.58	129	1338	52.63	5.26	15.79
20	31	117	88	0.24	301	0.96	167	382	81.48	7.41	25.93
21	38	129	57	2.64	722	2.7	122	9036	84.00	32.00	76.00
22	29	109	89	0.24	329	0.96	188	420	84.00	16.00	24.00
23	33	132	92						86.96	8.70	30.43
24	18	103	94						84.00	36.00	24.00
25	22	99	93						52.63	5.26	21.05
26	36	110	94	0.03		0.46	79.6	458	72.73	13.64	9.09
27	26	127	95						56.52	34.78	17.39
28	22	115	96						74.07	25.93	40.74
29	37	91	89	0.11		0.54	70.5	379	83.33	20.83	29.17
30	36	150	91	84.7					53.85	7.69	7.69
31	40	82	94	0.19	672	1.46	188	1425	76.92	15.38	15.38
32	32	87	74	1.44	698	1.02	191	9625	68.42	10.53	36.84
33	26	108	94			0.64	150	5523	42.11	5.26	21.05
34	36	106	84	0.18	537	1.52	216	1261	74.07	14.81	37.04
35	33	115	86	0.9	395	0.43	168	1719	64.71	17.65	35.29
36	26	130	96	0.1	485	0.72	143	1349	78.95	21.05	63.16
37	35	117	42	0.19	591	1.23	176	1355	94.12	11.76	58.82
38	24	90	88	4.46	573	1.64	161	4702	71.43	4.76	28.57
39	39	118	62	0.12	520	0.61	189	878	85.19	22.22	37.04

**Table 3:** Means, standard deviations, and Pearson correlations (r) for POCUS findings and outcomes POCUS, point-of-care ultrasound; RR; respiratory rate; HR, heart rate; CRP, C-reactive protein; LDH, lactate dehydrogenase *Correlation is significant at the 0.05 level (two-tailed). **Correlation is significant at the 0.01 level (two-tailed).

	Mean	SD	% Shred signs	% B-lines	% Merging B-lines	CRP	D-dimer	Ferritin	Procalcitonin	LDH	RR	HR	SpO_2_
% Shred signs	19.7	12.6	1.00	0.26	0.32*	-0.01	0.21	-0.04	-0.03	-0.04	-0.01	-0.09	-0.08
% B-lines	74.4	15.6	0.26	1.00	0.43**	-0.10	0.25	-0.09	-0.03	-0.05	0.28	-0.09	-0.38*
% Merging B-lines	29.1	17.8	0.32*	0.43*	1.00	-0.02	0.19	0.28	-0.08	0.13	0.28	-0.09	-0.36*
CRP	155.1	63.2	-0.01	-0.10	-0.02	1.00	0.07	0.16	0.24	0.33	-	-	-
D-dimer	1.7	2.2	0.21	0.25	0.19	0.07	1.00	0.31	0.89**	0.37	-	-	-
Ferritin	2046.7	2438.7	-0.04	-0.09	0.28	0.16	0.31	1.00	0.34	0.66**	-	-	-
Procalcitonin	6.2	22.3	-0.03	-0.03	-0.08	0.24	0.89**	0.34	1.00	0.44*	-	-	-
LDH	470.2	157.4	-0.04	-0.05	0.13	0.33	0.37	0.66**	0.44*	1.00	-	-	-
RR	30.3	7.8	-0.01	0.28	0.28	-	-	-	-	-	1.00	-0.03	-0.18
HR	111.3	18.5	-0.09	-0.09	-0.09	-	-	-	-	-	-0.03	1.00	-0.03
SpO_2_	85.6	13.8	-0.08	-0.38*	-0.36*	-	-	-	-	-	-0.18	-0.03	1.00

**Figure 3:**
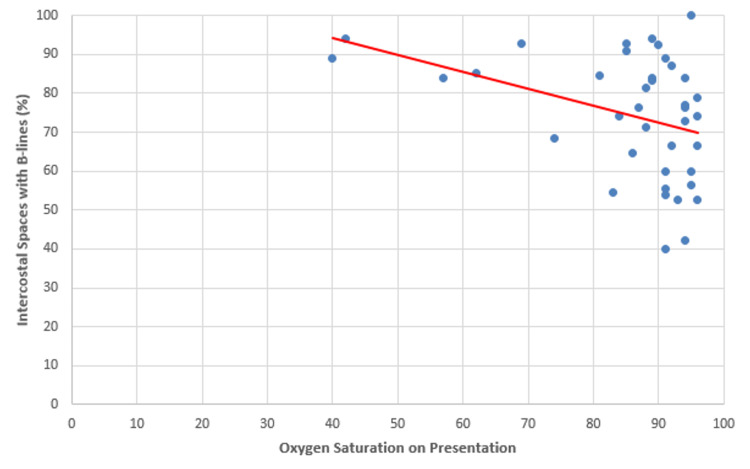
Percentage of intercostal spaces with B-lines versus oxygen saturation levels

The second characteristic of POCUS assessed was the relationship between the percentage of intercostal spaces with B-lines and the levels of acute phase reactants and vital signs as measured on presentation to the ED. A moderate negative correlation (r = -0.38) was found between the percentage of intercostal spaces with B-lines and oxygen saturation with a p-value of 0.02. As the percentage of intercostal spaces with B-lines increased, oxygen saturation on presentation decreased (Figure 3).

**Figure 4:**
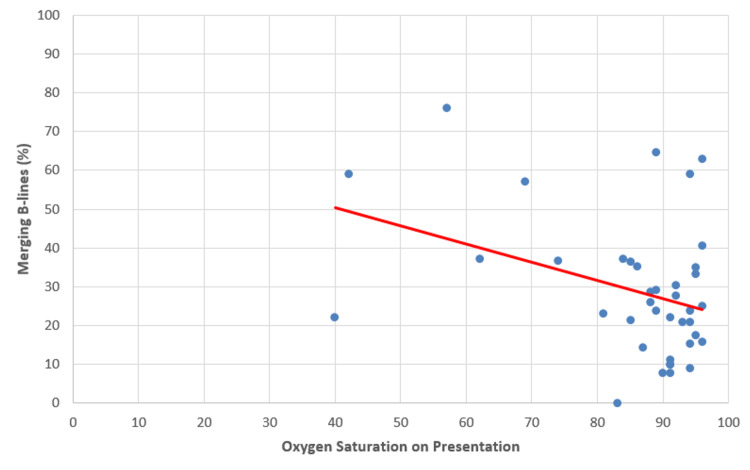
Number of merging B-lines versus oxygen saturation levels

Finally, the relationship between the percentage of merging B-lines and levels of acute phase reactants and vital signs as measured on presentation to the ED was assessed. A moderate negative correlation (r = -0.36) was found between the percentage of merging B-lines and oxygen saturation with a p-value of 0.02. As the percentage of merging B-lines increased, oxygen saturation decreased (Figure 4).

## Discussion

Both elevation of inflammatory markers and decreased oxygen saturation have been identified as drivers of morbidity and mortality in COVID-19 [14-16]. While our data did not show a correlation between sonographic findings in COVID-19 patients and inflammatory laboratory markers, it did demonstrate a correlation between POCUS findings of pneumonitis and worsening oxygen saturation. The presence of the shred sign did not show statistically significant correlation with lower oxygen saturation. The presence of B-lines in multiple anterior and posterior lung zones, as well as the presence of confluent B-lines in these zones suggested a lower oxygen saturation in our cohort. A higher number of B-line-positive lung zones was correlated with a lower oxygen saturation.

These findings add to the body of evidence in favor of the value of POCUS in assessing and managing patients with COVID-19 [17-20]. Among the several arguments for performing lung ultrasonography in the setting of COVID-19 infection, one is that lung ultrasound is performed at the bedside, by the treating clinician, without the need to move a potentially critically ill patient to the radiology suite [21]. Second, in the context of the current pandemic and the concern for the spread of a highly contagious illness, point-of-care imaging modalities are less likely to contaminate advanced imaging machines, such as CT scanners, rendering them inoperable for long periods of disinfection time [22]. Additionally, the sonographic assessment of the lung may be a viable alternative in resource-limited settings without access to other imaging modalities [23,24]. Lastly, lung ultrasound can likely play a role beyond diagnostic purposes in ventilation management for the critical COVID-19 patient [25].

Our study had a few limitations. This was a retrospective evaluation of patients in our institution. This type of study is inherently limited by confounders and selection biases. While we may show a correlation between factors in COVID-19 patients, causation cannot be determined from our data. This study may be limited by a lack of generalizability, with patients coming from a single, urban medical center with a patient population often lacking access to primary care resources. We did not adhere to a strict ultrasound scanning protocol, with a variety of intercostal spaces scanned in each patient (from 6 to 27 spaces in our 39 patients). While there was variability in the type of probe utilized for lung ultrasound scanning, this is in keeping with published data regarding the accuracy and quality of images obtained for the assessment of B-lines [26]. Furthermore, to reduce the effect of operator variability, all of the patients included had a minimum of four different lung views, ensuring clear views of intercostal spaces, both posteriorly and anteriorly. Given the novelty of COVID-19 pneumonia and the debate regarding optimal scanning conventions, we opted for a flexible sonographic approach as opposed to a rigid protocol [27-29]. This is also in keeping with the published expert consensus [30]. This may affect the likelihood of detecting pathology in the COVID-19 patient; however, leaving this at the discretion of the treating clinician is more consistent with real-world POCUS usage patterns.

Although this study was limited in scope, it is important in building on the growing body of research regarding the management and assessment of patients with COVID-19. Research into the novel coronavirus is limited by the very fact that it is still a fairly ‘novel’ virus. Potential areas of further research should include larger, prospective trials to determine how these POCUS findings correlate with patient disease course, morbidity and mortality.

## Conclusions

The outbreak of the COVID-19 pandemic has put emergency medicine physicians on the frontline of identifying and caring for patients affected by the virus. We aimed to assess the role of ultrasound as a diagnostic and prognostic tool regarding the novel coronavirus and to determine whether specific lung sonographic findings correlated with abnormalities in acute phase reactants or oxygen saturation. While assessing the percentage of intercostal spaces that were positive for the shred sign, it was found that the sonographic finding was not correlated with patients’ acute phase reactants or vital signs on presentation. However, both the percentage of intercostal spaces with B-lines and the percentage of merging B-lines were correlated with decreased oxygen saturation. No other statistically significant correlations were observed.
